# Enteric Nervous System and Its Relationship with Neurological Diseases

**DOI:** 10.3390/jcm13185579

**Published:** 2024-09-20

**Authors:** María José Luesma, Liberto López-Marco, Marta Monzón, Sonia Santander

**Affiliations:** 1 Department of Human Anatomy and Histology, University of Zaragoza, 50009 Zaragoza, Spain; 2 Department of Pharmacology, Physiology, Legal and Forensic Medicine, University of Zaragoza, 50009 Zaragoza, Spain

**Keywords:** enteric nervous system (ENS), gastrointestinal system, glial cells, central nervous system (CNS), neurological diseases, varicella zoster virus (VZV)

## Abstract

The enteric nervous system (ENS) is a fundamental component of the gastrointestinal system, composed of a vast network of neurons and glial cells. It operates autonomously but is interconnected with the central nervous system (CNS) through the vagus nerve. This communication, known as the gut–brain axis, influences the bidirectional communication between the brain and the gut. **Background/Objectives:** This study aimed to review neurological pathologies related to the ENS. **Methods:** To this end, a comprehensive literature search was conducted in the “PubMed” database. Articles available in “free format” were selected, applying the filters “Humans” and limiting the search to publications from the last ten years. **Results:** The ENS has been linked to various neurological diseases, from autism spectrum disorder to Parkinson’s disease including neurological infection with the varicella zoster virus (VZV), even sharing pathologies with the CNS. This finding suggests that the ENS could serve as an early diagnostic marker or therapeutic target for neurological diseases. Gastrointestinal symptoms often precede CNS symptoms, and the ENS’s accessibility aids in diagnosis and treatment. Parkinson’s patients may show intestinal lesions up to twenty years before CNS symptoms, underscoring the potential for early diagnosis. However, challenges include developing standardized diagnostic protocols and the uneven distribution of dopaminergic neurons in the ENS. Continued research is needed to explore the ENS’s potential in improving disease prognosis. **Conclusions:** The ENS is a promising area for early diagnosis and therapeutic development. Nevertheless, it is essential to continue research in this area, especially to gain a deeper understanding of its organization, function, and regenerative capacity.

## 1. Introduction

The enteric nervous system (ENS) is a division of the peripheral nervous system. It consists of a collection of glial cells and neurons, which originate in the neural crest and give rise to two concentrically distributed plexuses. These are the submucosal nerve plexus of Meissner (which provides innervation to the mucosa) and the myenteric plexus of Auerbach (where we can find, in turn, a subdivision called the deep myenteric plexus). The ENS has an integrative activity, thanks to an organized network of neurons that form different groups to manage the behavior of the gastrointestinal tract [1,2].

The enteric nervous system is involved in functions such as secretion, motility, mucosal maintenance, and immune defense mechanisms. It has a great capacity for regulation and coordination, as it allows the contents to be transported with precise timing, so that each part of the digestive tract can perform its function. This process is possible due to the existence of intrinsic primary afferent neurons, organized in the form of microcircuits, which integrate information and coordinate an appropriate response [2,3].

Unlike sympathetic or parasympathetic ganglion neurons, most enteric neurons do not receive direct innervation from the CNS, as they possess integrative neuronal activity [4]. However, bidirectional communication between the gut and the CNS is quite common. This communication is encompassed in the so-called gut–brain axis [1,5]. The most prominent connection is that of the vagus nerve. A total of 90% of the vagal fibers between the gut and the brain are of the afferent type, which could mean that the brain is a receptor for the communication of the gut–gut axis. Through these channels, the CNS can learn about the composition of the contents and the presence or absence of hunger [3].

Stimulation of the CNS by the gut influences learning, hippocampal memory, motivation, and emotions. There is a great diversity of neuronal and glial subtypes, as well as neurotransmitters, that are superior to those of the CNS. These systems depend on a functional interaction with glial cells in the ENS, being essential for neuroprotection, neural maturation, synapse formation, and the creation and destruction of neurotransmitters [6,7,8].

Classically, the ENS has been classified as part of the peripheral nervous system; however, due to its organization, functional independence, and neuroactive substances it contains, it is much more similar to the CNS [4,9]. In fact, the ENS has been implicated in some neurological diseases such as autism spectrum disorder, amyotrophic lateral sclerosis, transmissible spongiform encephalopathies, Parkinson’s disease, Alzheimer’s disease, Huntington’s disease, and varicella zoster virus infection.

There are some model studies describing common pathophysiological mechanisms. This could be the reason for the higher frequency of gastrointestinal comorbidity in these diseases [10,11,12,13,14]. The importance of this fact lies in the fact that nerves that interconnect the ENS with the CNS could serve for the propagation of neurological diseases [5,11]. The complex relationship between inflammatory bowel diseases (IBDs) and neurological and psychiatric disorders underscores IBD as a significant comorbidity affecting mental health. Emerging research highlights the role of melanocortins in IBD pathogenesis, particularly through their receptors, MC3R and MC5R, which are linked to disease activity. Targeting these receptors may present new therapeutic opportunities for IBD management [14].

The ENS represents a study structure that can simulate CNS pathophysiology, thanks to their similarities in structures, functions, molecular signaling pathways, and shared pathologies [9,11,12].

The primary aim of this study is to carry out an in-depth bibliographic search of neurological pathologies in which the ENS is involved in one way or another. The fact that certain gastrointestinal symptoms manifest themselves much earlier than those affecting higher CNS structures and the greater accessibility of the ENS to the CNS make the ENS an interesting structure both from the point of view of the study of the physiopathology of neurological diseases and their diagnosis and even as an alternative therapeutic target for them that constitute our secondary outcomes.

## 2. Materials and Methods

The study used a multi-step methodology as follows:

1. Initial Search: A general search with the term “enteric nervous system” with the “human” filter yielded 4589 publications in PubMed. Twenty articles were included for a comprehensive understanding, including general data on the embryology of the enteric nervous system, the enteric nervous system in adults, and the relationship between the enteric nervous system and the microbiota. This step was carried out between 1 December 2023 and 31 January 2024.
2. Systematic Search: To begin the study, a search was conducted on PubMed to quantify the amount of literature describing the enteric nervous system and its relationship with neurological diseases. The term “enteric nervous system AND neurological diseases” was used with the filter “Humans”. A total of 224 articles were obtained. After conducting the initial search on the enteric nervous system and the most common neurological pathologies, terms related to neurological pathologies (frontotemporal dementia, amyotrophic lateral sclerosis, spongiform encephalopathy, Parkinson disease, Alzheimer disease, multiple sclerosis, autism spectrum disorder, varicella zoster virus) were combined with ‘enteric nervous system’ in a systematic search. Using the Boolean operators AND and OR, the most appropriate combination of terms was created to yield the best results. The combination was as follows: (enteric nervous system AND neurological disease) OR (frontotemporal dementia AND amyotrophic lateral sclerosis)) OR spongiform encephalopathy) OR Parkinson disease) OR Alzheimer disease) OR multiple sclerosis) OR autism spectrum disorder) OR varicella zoster virus. A total of 93 results were obtained in PubMed. Before proceeding to the selection of articles, the inclusion and exclusion criteria were defined as follows:
  - Inclusion criteria: Any paper related to any article related to neurological pathology associated with the enteric nervous system in humans, including studies, reviews, case series, editorials, and guidelines published in the last 10 years in English or Spanish.
  - Exclusion criteria: Unusual manifestations, neurological diseases not related to enteric nervous system, those studies older than 10 years, and, finally, pathology in animals.

A total of 73 articles were obtained after applying inclusion and exclusion criteria; 56 articles were selected for further analysis. Seventeen articles were discarded for not adding relevant information. This step was conducted between 1 February 2024 and 31 March 2024.

Other bibliographic sources were consulted. In the Cochrane database, 10 reviews were found that included the enteric nervous system and neurological disorders. However, after reading the abstract of each, they were not included in this review.

3. Manual Search: Based on references from the selected studies, 5 additional articles were included, bringing the total to 61 empirical articles published between 2013 and 2024. These were articles obtained from other databases such as “Google Scholar”, “Sci-Hub”, or “Elsevier”. This step took place during the month of May 2024.

**Figure 1:**
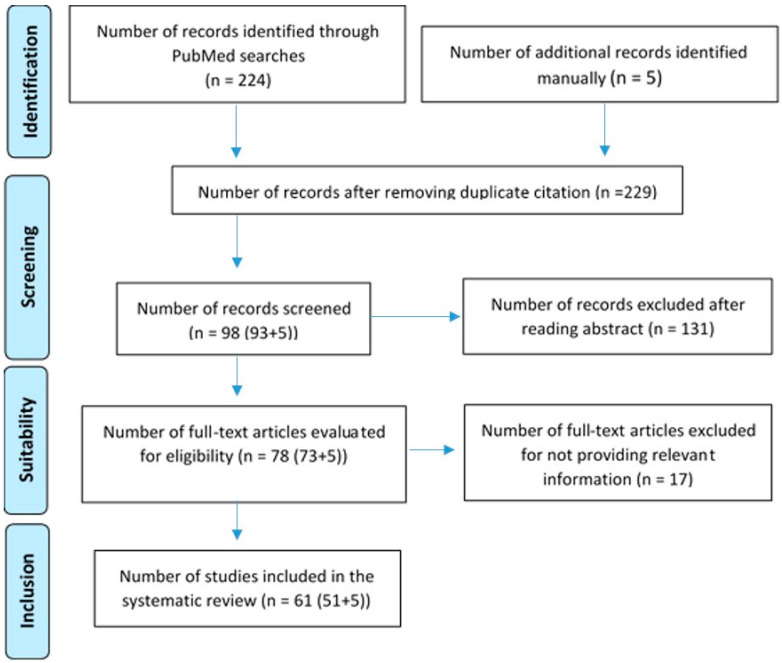
Modified PRISMA flowchart [13,15].

The study’s systematic review adhered to PRISMA guidelines, focusing on human-related neurological diseases associated with ENS. This study was conducted according to the Preferred Reporting Items for Systematic Reviews and Meta-Analyses (PRISMA) statement [13,15]. The protocol has been registered in the PROSPERO database (registration number: 592407). The PRISMA flowchart below (Figure 1) summarizes the search process.

Two of the authors of this paper acted as independent reviewers, conducting the literature search, study selection, and assessment of methodological quality. The participation of two reviewers ensured a comprehensive review and minimized potential biases in the identification and selection of the included studies. Additionally, the third author, also a reviewer, played the role of a mediator in case of disagreements between the reviewers.

As a tool, Mendeley’s (Mendeley (2022), Mendeley-Reference Management Software (Mendeley Desktop 1.19.8) Research Network, https://www.mendeley.com/ (accessed on 25 January 2021)) was used as a bibliographic reference manager, enabling the organization and management of references from studies identified during the search. Regarding the assessment of study quality, an approach based on the authors’ criteria was followed, aligned with the 16 items specified by the AMSTAR-2 (A Measurement Tool to Assess Systematic Reviews) tool.

## 3. Results

In the results, some pathologies are presented such as diseases with prion-like behavior, pathologies involving movement disorders, cognitive and behavioral dysfunctions, and herpes zoster virus (HZV).

It is worth noting the disproportion of information between the different diseases in the literature. On the one hand, the prevalence of neurological diseases varies significantly, with many more studies on frequently occurring diseases. On the other hand, the discovery of the relationship between ENS and certain neurological diseases is very recent, resulting in few studies, while in other cases, this relationship has been known and investigated for much longer.

**Figure 2:**
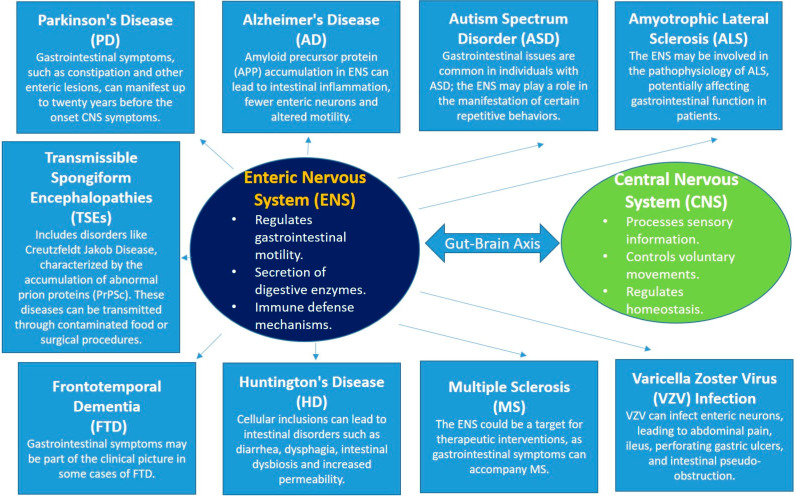
This comprehensive approach illustrates the various neurological diseases linked to the ENS.

Figure 2 illustrates the various neurological diseases linked to the ENS, emphasizing the potential for the ENS to serve as an early diagnostic marker and therapeutic target across multiple conditions.

### 3.1. Transmissible Spongiform Encephalopathies

Prion diseases represent a group of neurodegenerative disorders caused by protein misfolding. Their pathophysiology is based on the accumulation of endogenous cellular prion proteins which, when misfolded, trigger a marked pathogenicity in cells and tissues [3,10,15,16,17] leading to progressive deterioration leading to neurodegeneration and ultimately to the death of the individual [10].

Among the most recognized prion diseases in humans is Creutzfeldt–Jakob disease (CJD), a type of transmissible spongiform encephalopathy, the origin of which can be attributed to both familial variants, characterized by inherited mutations in the PRNP gene such as the E200K mutation, and acquired forms [1]. In all of them, there is an accumulation of pathological cellular prion protein (PrPC), which is transformed into an abnormal protein (PRPSc), showing transmissible behavior throughout the CNS [16].

In the context of spongiform encephalopathies, ENS is recognized as the main route of entry of the causative pathogen [10], mainly through the ingestion of food contaminated by the pathological protein. Another route of entry of these prion glycoproteins is the performance of invasive surgical procedures with material from affected patients [17].

Entry of these pathological proteins could be via the following methods: through the immune system, a bridge between the gut and the brain in the case of ingestion; through M cells acting as vectors to translocate prion proteins to lymphoid cells; or through receptors for cellular prion proteins on the brush border of enterocytes [17].

Both glia cells and enteric neurons have receptors for the prion protein, which could make them susceptible if ingested, as they are in close proximity to the intestinal lumen [3,10,17].

### 3.2. Parkinson’s Disease

Parkinson’s disease (PD) is characterized by a progressive degeneration of nigrostriatal dopaminergic neurons. PD belongs to the α-synucleinopathies [10] and results in movement disorders. Common symptoms include rigidity, resting tremor, postural instability, and bradykinesia [10].

The α-synuclein protein is expressed in both the CNS and the ENS. It plays a crucial role in neuronal survival, as its functions include interaction with the dopamine transporter, regulation of neurotransmitter release in synaptic vesicles, and involvement in neuronal apoptosis processes [10,12].

In PD, α-synuclein forms protein aggregates that are resistant to proteases. This process leads to the formation of Lewy bodies, a characteristic pathological lesion of the disease. In addition, misfolded α-synuclein has the ability to spread from cell to cell, similar to the action of a prion. When an α-synuclein is taken up by another neuron, it can be used as a template for the pathological folding of other α-synucleins triggering aggregates, which ultimately produce Lewy bodies [12,18]. Animal models describing α-synuclein alterations demonstrate prolonged gastrointestinal transits and altered colonic motility [18].

In this disease, there is evidence of an overstimulation of the innate immune response and dysregulation of its signaling. Part of the alteration in this immune response could come from the Toll-like receptors (TLRs). This pathological immune response is responsible for generating modifications in the gut microbiota, for increasing inflammation both locally and systemically, and for triggering enteric neuroglial activation leading to the development of α-synuclein-associated pathology [11,19,20,21,22,23]. TLR activation promotes inflammation and oxidative stress, suggesting that their use as a therapeutic target could improve gut health, restore epithelial barrier function, and mitigate excessive immune response, thus preventing the neurodegenerative process [24].

Three decades ago, abnormalities in the ENS were documented in PD patients. Dopaminergic neurons in the ENS are necessary for proper gastrointestinal motility. In animal models, the ENS has been shown to be vulnerable to PD. For example, systemic administration of MPTP (1-methyl-4-phenyl-1,2,3,6-tetrahydropyridine) causes nigrostriatal damage and gastrointestinal dysfunctions such as hypersalivation, dysphagia, delayed gastric emptying, nausea, constipation, and altered bowel habits [10].

In this entero-neuronal circuit, one could highlight the importance of entero-endocrine cells, cells sensitive to chemical alterations resident in the mucosa of the gastrointestinal tract. These cells are involved in responding to signals from nutrients or the presence of bacteria in the lumen of the gut [12,18,19]. Enteroendocrine cells have neuron-like characteristics (they have neurotrophin receptors, pre- and postsynaptic proteins, and processes similar to those carried out in axons) and are capable of producing dopamine. There is a possibility that enteroendocrine cells come into contact with nerves, so that there is a neural circuit connecting the intestinal lumen with the nervous system. These cells may undergo accumulations of α-synuclein, so their transmission to enteric neurons could be the first step in the cascade leading to the development of PD [12,18], suggesting that damage to the ENS would precede the onset of CNS symptoms [12,19,24].

Transmission of PD from the gastrointestinal tract to the brain via the vagus nerve is a possibility, although it has not been confirmed as the sole cause. It has been observed that up to 7% of affected patients have no α-synuclein inclusions despite extensive nigrostriatal involvement. In this context, it has been documented that those patients undergoing truncal vagotomy, a surgical procedure involving section of the vagus nerve, have a lower risk of PD progression [25]. However, further analysis of this study suggests that these findings are not entirely conclusive [26].

Despite evidence of prion behavior of α-synuclein in post-mortem observations, not all studies consider this relationship strong enough to be a therapeutic target [18,19].

### 3.3. Huntington’s Disease

Huntington’s disease (HD) is an inherited neurological disease caused by a mutation in the HTT gene. This mutation involves a CAG trinucleotide repeat, which translates into small proteins that when accumulated produce cellular inclusions, causing neurodegeneration and other dysfunctions such as motor and psychiatric disorders and intestinal disorders such as diarrhea, dysphagia, intestinal dysbiosis, and increased permeability [27,28].

HD mainly affects neurons with increased labeling for the neurotransmitter gamma-aminobutyric acid (GABA). Alterations in the HTT gene have also been found to be present in neurons of the ENS, suggesting that the weight loss associated with this disease could be attributed to two main causes: gut dysfunction due to significant neurodegeneration and marked alterations in the gut microbiota [28].

The main cause of this dysfunction in the ENS is due to the loss of neuropeptide production capacity. However, alterations in fluid and solid intake behavior due to associated hypothalamic abnormalities have also been identified [27].

### 3.4. Amyotrophic Lateral Sclerosis and Frontotemporal Dementia

Amyotrophic lateral sclerosis (ALS) and frontotemporal dementia are two neurodegenerative diseases that share a genetic basis and forms of pathology that can affect the ENS. These pathologies are associated with alterations in cytoplasmic nuclear transport at the cellular level, with TDP-43 cytoplasmic inclusion bodies appearing in motor neurons. ALS is a neurodegenerative disease characterized by selective involvement of motor neurons in the brainstem, spinal cord, and motor cortex. The disease degenerates into muscle atrophy and paralysis of limb and respiratory muscles [10,27].

Some of the mutations associated with these diseases are alterations in TARDBP (encoding DNA-binding protein 43, TDP-43), C9orf72, glial cell line-derived neurotrophic factor (GDNF), and FUS (gene related to TDP-4 and familial ALS) [10].

The mutation of TARDBP in TDP-43 transgenic mouse models (expressed in glial cells and enteric neurons) causes symptoms of intestinal obstruction and sudden death. In addition, they are associated with slowing of the gastrointestinal tract and sluggish colonic motility [29].

ALS has two variants, familial and wild type. Alterations in the FUS protein are associated with very aggressive and juvenile diseases, and the C9orf72 alteration is also associated with familial ALS. All these alterations highlight the importance of misfolding of proteins associated with familial ALS. However, no studies were found linking FUS and C9orf72 alteration in human or animal ENS [27].

Another alteration that could be related is that of the enzyme superoxide dismutase 1 (SOD1), which alters the binding between enterocytes, resulting in increased mucosal permeability. Disruption of SOD1 leads to alterations in the proliferation and differentiation of the mucosal epithelium, including cholinergic and serotonergic neurons. In addition, survival signals of enteric neurons are altered, contributing to the development of the disease. However, whether pathology outside the CNS can be transmitted to the brain is under investigation [6].

In the case of the GDNF alteration, we can highlight that it is a transforming growth factor ß that promotes the survival of motor neurons and mesencephalic dopaminergic neurons. This intracellular signaling pathway uses a specific receptor, the tyrosine kinase receptor C-RET, a product of the RET proto-oncogene, which is altered in ALS. C-RET is not expressed in all tissues; however, one of the tissues where it is expressed is the ENS, which is vital for the survival of enteric neurons [1,6].

C-RET is expressed in the embryological development of many CNS and peripheral neuronal subsets, making it essential for postnatal neuronal survival of dopaminergic neurons and nerve plexuses throughout the intestinal tract (C-RET is positive in the myenteric and submucosal ganglia to a lesser extent). In ALS, neurons lose survival signaling pathways; however, some capacity for tissue repair using embryonic developmental cellular pathways has previously been described. These indications lead to the conclusion that there is a pool of multipotent stem cells that could differentiate into mature cells [1,6,27].

All these alterations open the door to the possibility of further study to find a therapeutic target for this infamous disease. With this evidence, it is clear that genetic alterations in ALS and frontotemporal dementia may be associated with ENS disorders. It is necessary to continue research on the ENS as a potential therapeutic target, the key being to find the differences that allow for the regenerative capacity and maintenance of the structure of enteric neurons versus CNS neurons [6].

### 3.5. Multiple Sclerosis

Multiple sclerosis (MS) is a degenerative neurological disease characterized by chronic demyelination of the CNS. Histologically, demyelinating plaques appear, and their origin has been linked to Epstein–Barr virus infection and HLA-DR15. The presence of these coadjuvant factors may trigger a specific reaction of T-CD4+ lymphocytes to myelin antigen [30]. In MS patients, the following has been found [31]:

- Increased intestinal permeability. Higher rate of inflammation and increased expression of haptoglobin precursor protein 2 [32].
- Alterations of the ENS. There are structural (increased gliosis and decreased enteric neurons) and functional abnormalities [33].
- Higher prevalence of altered microbiota. There is a difference between the intestinal microbiota of MS patients and healthy subjects [22,34,35,36,37,38,39]. Among the most notable results, the higher the frequency of polysaccharide A from the capsule of the bacterium *Bacteroides fragilis*, the lower the inflammatory activity. This antigen is a potent activator of immune cells, capable of inducing clonal expansion of CD4+ T cells and increased secretion of IL-10 in T and B lymphocytes, and its immunomodulatory and protective role in the development of MS has been described [37,38,40].
- Elevated levels of short-chain fatty acids, bile products, and other metabolites. An increased presence of microbial metabolites has been found. Short-chain fatty acids appear to play a prominent role, being able to cross the blood–brain barrier and control neuroimmune homeostasis [41]. Another metabolite of note is acetate. It has been reported that elevated acetate levels in people with MS compared to control subjects correlated with a higher disability status scale score and a higher prevalence of CD8+ T cells [42].

Therefore, alterations in the gut–brain axis have been demonstrated in MS patients, suggesting the involvement of the ENS in the pathophysiological process of the disease, which may be a therapeutic target [9,31].

### 3.6. Alzheimer’s Disease

Alzheimer’s disease (AD) is defined as a neurodegenerative pathology characterized by a progressive deterioration of memory and cognitive functions, manifesting a clinical picture of dementia. In this disorder, an accumulation of extracellular plaques containing beta-amyloid (Aβ) and the presence of intracellular neurofibrillary tangles of hyperphosphorylated tau protein are observed [10]. Nowadays, it is suggested that the brain is connected to the gastrointestinal tract, especially the enteric nervous system and gut microbiome. Studies have found a positive association between AD and gastrointestinal diseases [43].

In the ENS, the amyloid precursor protein is required for functions such as motility, immunity, and gastrointestinal secretion. In addition, most neurons in the ENS are cholinergic, which would theoretically imply AD involvement in the ENS as they have a similar structure. However, the ENS does not show disease-associated neurodegeneration, which may be due to its ability to maintain structure [44]. In animal models, it was found that those with amyloid precursor protein (APP) accumulation had a greater tendency to intestinal inflammation, fewer enteric neurons, and altered motility. In turn, the greater the amount of intestinal inflammation, the greater the deposition of plaques in the CNS [10,45].

It has been shown that after intestinal inoculation of Aβ oligomers, the Aß accumulation pathology spread not only within the cholinergic neurons of the ENS (causing dysfunction), but also ascended through the vagus nerve causing cognitive defects [27,43].

In addition, Lin (2018) conducted a study to assess the incidence of AD in patients with truncal vagotomy, concluding that those undergoing truncal vagotomy had a lower risk of dementia [46].

The presence of Aß plaques in the intestinal submucosa has also been reported in humans. However, due to the high prevalence of this neurodegenerative pathology, it is more than necessary to explore the possibility of a potential link between it and ENS [27].

Gut dysbiosis may contribute to disease development and progression. Microbiota-gut–brain communication can impact neurodegenerative diseases through various mechanisms, including the regulation of immune function, the production of microbial metabolites, and the modulation of host-derived soluble factors [22,23].

### 3.7. Autism Spectrum

Autism spectrum disorder (ASD) is a clinical condition characterized by a neurodevelopmental disorder that is usually diagnosed in childhood due to specific behavioral manifestations. Distinguishing features include social difficulties, communication deficits, and the presence of repetitive patterns of behavior [10].

Studies have found a higher incidence of gastrointestinal symptoms in children with ASD, up to four times more compared to the general population [47]. These symptoms are often considered as comorbidities. Currently, no specific diagnostic marker for autism has been identified. Furthermore, it is recognized that within ASD, there is a diversity of mental disorders that manifest with different phenotypic profiles [48].

It is estimated that between 100 and 800 genes could be related to autism. From the evidence available in the scientific literature, it is argued that a fraction of ASD cases is associated with alterations in the ENS [49,50,51].

In the search for greater diagnostic accuracy, the implementation of a reverse phenotyping process is proposed, which involves the identification of altered genes to define the corresponding phenotype [10,48]. The following are examples:

- Abnormal expression of the gene encoding CHD8 leads to alterations in enteric neurogenesis together with slow gastrointestinal transit. This description indicates that gastrointestinal disturbances are not comorbidities, but part of the phenotype of autism spectrum disorder.
- Transcription factor 4 (TCF4) haploinsufficiency in Pitt–Hopkins syndrome presents with alterations of rectal motility and upper gastrointestinal transit.
- Disruption of the SLC6A4 gene encoding the sodium-dependent serotonin (5-HT) transporter is associated with autistic behavior disorder, together with SNE hypoplasia and slow gastrointestinal tract.

Patients with ASD often manifest digestive symptoms such as abdominal pain, stool retention, and bulky stools. These disturbances are related to the level of serotonin (5-HT) in the blood, which is mostly derived from the gut. In addition, up to one-third of patients affected by this disorder have elevated serotonin levels. This could be the reason for these digestive symptoms [10,47].

Agents that cause congenital damage to the CNS may have a similar effect on the ENS. In the case of valproic acid, epidemiological studies have shown a link between prenatal exposure to the antiepileptic drug and increased risk of ASD and intestinal inflammation [52].

The leaky gut theory has been proposed as part of the pathophysiology of autism spectrum disorder. It is suggested that alterations in the intestinal epithelial barrier may trigger inappropriate signaling by luminal components, affecting the relationship with bacteria, toxins, and macromolecules. Intestinal permeability is altered in children with ASD, being pathological in 35% [53].

Subjects infected with the bacterium *Bacteroides fragilis* were shown to restore their intestinal epithelial barrier integrity, resulting in improved communication, exploration of the outside world, sensory motor impairment, and cessation of repetitive activities. These findings suggest that certain symptoms of ASD may be reversible. Thus, restoration of gut microbiota and barrier integrity may improve some social behaviors [20,39,54].

### 3.8. Varicella Zoster Virus ENS Viral Infection

Neurological infection with varicella zoster virus (VZV) occurs in childhood disease with the clinical form of varicella, a mild to moderate disease that allows the virus to gain access to neurons where it remains permanently. The VZV remains mainly in the dorsal root ganglia and cranial nerve ganglia [17]. This virus can subsequently reactivate as a secondary infection, especially in individuals with a weakened immune response, resulting in pain and excessive skin sensitivity. The clinical presentation commonly consists of a vesicular rash, which facilitates diagnosis [10].

However, it has recently been reported that the virus also establishes latency in enteric neurons in virtually all patients who have had chickenpox. In fact, in cases of severe immunodeficiency, enteric neurons may be destroyed, triggering ileus [55].

When reactivated in neurons of the ENS, they do not present cutaneous symptoms, so the difficulty of diagnosis is high. Enteric zoster is a cause of perforating gastric ulcers, unexplained abdominal pain, and intestinal pseudo-obstruction due to acquired agangliosis (similar to Chagas disease) [55]. One way to diagnose VZV is by detection by a non-invasive saliva test (VZV DNA appears in saliva when an infection occurs somewhere in the body). A clinical trial showed that in patients with chronic abdominal pain who tested positive for suspected enteric zoster, treatment with valacyclovir was followed by the disappearance of abdominal pain in 100% of patients [56]. It could be concluded that this test in a patient with abdominal pain with no cause found after an extensive gastroenterological examination could be an indication for the diagnosis of enteric zoster [56].

With the aging of the population and the increased use of immunosuppressive therapy, the incidence of zoster has increased considerably. Studies in developed countries have shown that enteric zoster is not as marginal as previously thought, so it is important to be aware of this clinical situation in order to solve this pathological condition. In addition, it has been mentioned that VZV may be involved in irritable bowel syndrome, gastroparesis, and inflammatory bowel disease, but again, there is still a lack of literature on the topic to address this issue [10].

**Figure 3:**
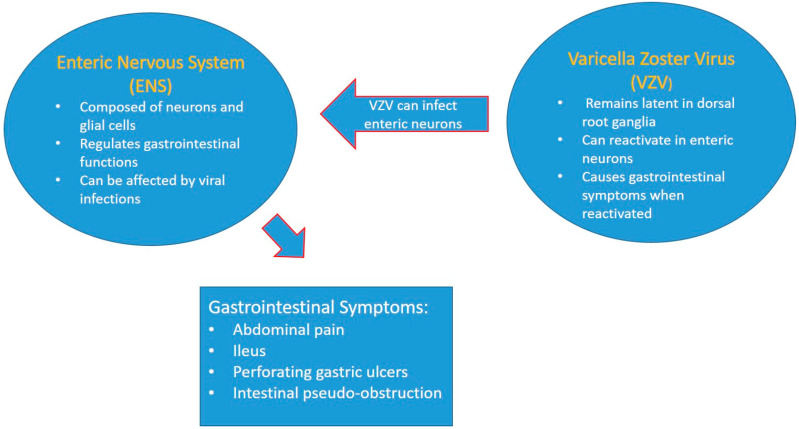
This figure visually represents the relationship between the ENS and VZV, highlighting how VZV can affect the ENS and lead to various gastrointestinal symptoms.

Figure 3 visually represents the relationship between the ENS and VZV, highlighting how VZV can affect the ENS and lead to various gastrointestinal symptoms.

## 4. Discussion

After reviewing the relationship between neurological diseases and the ENS, we can state that the ENS can become an opportunity to address some neurological pathologies. The ENS can be involved in many different ways in diseases, from being the gateway to neurological conditions, as in the case of spongiform encephalopathies or PD [12,17,18,19], to being part of the clinical picture, as in the case of some types of ASD, ALS, frontotemporal dementia, HD, and AD [27,28].

In turn, SNE could be used as a diagnostic and therapeutic target for some diseases, such as enteric VZV infection, Eo, MS, or to improve certain repetitive behaviors in ASD [25,54,56].

With regard to PD, it has been found that the ENS could be useful for early diagnosis, prevention, and treatment. The ENS is a much more accessible structure than the CNS; the fact that the characteristic lesions of this pathology appear much earlier than the neurological symptoms make it a structure of great interest for early diagnosis strategies [18].

Studies in rodents have shown that the histological lesion that appears first in PD is the progressive accumulation of α-synuclein in enteric neurons, then in the dorsal motor nucleus of the vagus, and finally in the substantia nigra [25]. Some investigations have proposed biopsies for colonoscopies, since 150 submucosal neurons and thousands of nerve fibers can be obtained. This procedure would be a realistic possibility for early diagnosis of the disease. It is based on the fact that Lewy bodies are more numerous in patients with PD than in the control groups and that the expression of enteric α-synuclein was found unanimously in all patients [18]. The possibility of early diagnostic histological markers of α-synuclein aggregates was explored as an early diagnostic marker. The idea was to take an enteric lymph node sample in the absence of CNS symptoms and observe for protease-resistant α-synuclein aggregates [12,18,19]. However, its use as a differential marker for healthy and diseased controls posed problems, as up to 50% of healthy controls had aggregates of this protein [18]. It has been shown that the myenteric plexus is more frequently affected than the submucosal plexus, so even if these biopsies are not routinely taken, it is still much easier to access these plexuses than to take biopsies from the CNS itself [1,11,19]. With the proposed arguments, it can be admitted that optimizing this diagnostic process would be very beneficial, since in PD patients, intestinal lesions appear up to twenty years earlier than CNS manifestations.

The main problem we encounter with regard to establishing an early diagnosis of some neurological diseases is the lack of benefit in the final prognosis of the disease, which implies generating unnecessary suffering [57]. However, it has been shown that exposure to environmental toxins could lead to degeneration of the nigrostriatal pathways, resulting in the accumulation of pathological proteins that constitute Lewy bodies [11,12,19]. Thus, in subjects who have been exposed to these toxins and who, through adequate information on the risks and benefits of this information, decide to accept it, the following hypothesis of early diagnosis could be put forward [1,12,19]: The procedure consists of a colonoscopy in which samples of enteric neurons are taken to check for Lewy body accumulation as a marker of PD [58,59,60]. A simple detection of α-synuclein does not seem to be as specific for the disease, as it may be found in up to 50% of healthy controls [58].

Given these findings, it would be reasonable to perform such a colonoscopy [59]. However, we are faced with a number of difficulties in putting it into practice:

- The first difficulty is to develop a protocol in which pathologists would establish histological lesion criteria suggestive of pathology. By standardizing morphological immunostaining patterns with the use of autoantibodies to improve the prognostic values of α-synucleinopathies and Lewy bodies, their presence does not always necessarily imply disease [18,19].
- The second difficulty is the irregular distribution of dopaminergic neurons in the ENS, which may hinder their adequate sampling, as the organization of the ENS is currently not fully understood. If we need to biopsy those neurons, especially dopaminergic neurons because they have the best diagnostic results, we would need more certainty in localization [7,18,61].
- The third difficulty is that the analysis of biopsies, although it may have a high diagnostic value, as histological lesions can be observed up to 20 years before the first symptoms of the disease, has no prognostic value for disease activity or progression [57].

For these reasons, a better understanding of the organization and characterization of the ENS is necessary for its possible use as an early diagnosis of the disease.

On the other hand, current evidence suggests that the gastrointestinal therapeutic target could be used for the treatment of PD comorbidities [25], allowing improvement of intestinal inflammation and permeability. Examples of proven treatments include the following [18,25]:

- Certain dietary interventions have shown beneficial results in modulating microbiota dysbiosis, reducing intestinal permeability, and decreasing oxidative stress and inflammation. One example is polyunsaturated fatty acids such as the omega-3 fatty acid docosahexaenoic acid (DHA). The results of this intervention led to a reduction in motor symptoms due to improved mitochondrial dysfunction.

Another reported benefit was the reduced accumulation of α-synuclein. The study highlighted the importance of taking probiotics, which increases patient health by creating a more favorable gastrointestinal environment and performing other functions, such as modulating immune response, decreasing intestinal permeability, and alleviating complications. In turn, probiotic administration enhances levodopa absorption, improving behavioral and cognitive performance [25].

Altering TLR activity and expression could reduce or prevent the development of PD. Currently, none of the treatments for the disease produce alterations in the progression of gastrointestinal symptoms, so the combination with modulators could prevent dysfunction and gastrointestinal symptoms, positively influencing dysbiosis and protecting neuroglia [11].

Truncal vagotomy [31]. Although not entirely clear, it may increase protection against PE. Other alternatives to the use of the ENS for the treatment of CNS pathologies include the following:

In ASD, infection with the bacterium *Bacteroides fragilis* restores the integrity of the gut barrier resulting in an improvement of ASD-like behaviors. Therefore, not all CNS alterations are irreversible and symptomatology can be improved by optimizing gut flora and gut barrier permeability [62].

The use of probiotic supplements in MS has been shown in three clinical trials to modulate the inflammatory response through the intake of *Lactobacillus* and *Bifidobacterium* [63,64], as it has been shown to restore the integrity of the intestinal barrier and act on immunoregulatory pathways of inflammation. Its daily application results in lower rates of disability and anxiety and lower levels of IL-8 and CD-80 on peripheral monocytes and TNF [65,66,67].

VZV disease is an underdiagnosed pathology that can present with painful chronic dyspepsia. In patients with a combination of immunosuppression and gastric ulcers, long-standing abdominal pain, and/or intestinal pseudo-obstruction, DNA sequencing of VZV saliva would be considered as a diagnostic procedure for viral infection. If positive, treatment with valacyclovir would be proposed. A 100% efficacy was demonstrated in the Gershon AA clinical trial [56].

Table 1 summarizes the main relationships described between ENS and neurological diseases.

There is still relatively little literature on the ENS and its relationship with neurological diseases. It has been described many times that the ENS has a greater capacity to preserve its structure despite severe neurological diseases such as ALS, frontotemporal dementia, and AD [1]. The key to why the ENS is less affected regardless of the extent of the primary disease may be related to the greater capacity for maintenance and regeneration of the internal structure of this system [44,67]. Exploring these mechanisms could be of great help in advancing the study of neurological diseases affecting the CNS.

It is important to mention that, although paradoxically the ENS is classified within the PNS, it is more similar in structure and function to the CNS. However, it has a greater capacity for regeneration. Some of the processes of its formation, organization, and regeneration are unknown, and clarification of all these aspects could lead to finding therapeutic targets that improve the final prognosis of these diseases [44].

## 5. Conclusions

The ENS is affected in neurological conditions such as PD, autism, ALS, frontotemporal dementia, and AD including neurological infection with VZV. The ENS emerges as a structure of interest for developing early diagnostic strategies, especially in PD due to the progressive accumulation of Lewy bodies in enteric neurons prior to CNS involvement. Although there are promising possibilities for early diagnosis, such as performing colonoscopies to obtain biopsies of the ENS, there are difficulties related to the development of diagnostic protocols, the irregular distribution of dopaminergic neurons, and the analysis of the biopsies obtained. On the other hand, alternative therapeutic approaches using the SNE have been identified to address diseases such as PD, ASD, and MS, including specific dietary interventions and Toll-like receptor (TLR) modulators. In VZV disease, DNA sequencing of VZV saliva would be considered as a diagnostic procedure for viral infection. The ENS is a potential therapeutic target for the treatment of neurological diseases; however, further research in this field is needed, especially to better understand its organization, function, and regenerative capacity.

## Figures and Tables

**Table 1:** Contributions of the SNE to neurological diseases.

Diseases	Contributions of the Enteric Nervous System
Parkinson’s disease	Early diagnosis: taking biopsies by colonoscopy Treatment: dietary interventions, modulation of the TLR response, and vagotomy
Alzheimer’s disease	Vagotomy as a protective element against dementia
Transmissible spongiform encephalopathies	SNE as a possible entry point
Multiple sclerosis	Treatment with probiotic supplements that modulate the autoimmune response
Shingles virus	The use of the saliva test for the detection of virus DNA in enteric zoster cases
Autistic spectrum disorder	Treatment of repetitive behaviors by *Bacteroides fragilis*

## Data Availability

Data sharing is not applicable to this article.
